# A Low-Cost Energy-Efficient Cableless Geophone Unit for Passive Surface Wave Surveys

**DOI:** 10.3390/s151024698

**Published:** 2015-09-25

**Authors:** Kaoshan Dai, Xiaofeng Li, Chuan Lu, Qingyu You, Zhenhua Huang, H. Felix Wu

**Affiliations:** 1 State Key Laboratory of Disaster Reduction in Civil Engineering, Tongji University, 1239 Siping Rd., Shanghai 200092, China; E-Mails: kdai@tongji.edu.cn (K.D.); lixiaofeng168@yeah.net (X.L.); 2 State Key Laboratory for GeoMechanics and Deep Underground Engineering, Xuzhou 221008, China; 3 State Key Laboratory of Petroleum Resources Research, Institute of Geology and Geophysics, Chinese Academy of Sciences, Beijing 100029, China; E-Mail: luchuan09@126.com; 4 University of North Texas, 3940 N. Elm Street F115M, Denton, TX 76207, USA; E-Mails: zhenhua.huang@unt.edu (Z.H.); felix.wu@unt.edu (H.F.W.)

**Keywords:** cableless geophone, surface wave, low cost, energy consumption, validation test

## Abstract

The passive surface wave survey is a practical, non-invasive seismic exploration method that has increasingly been used in geotechnical engineering. However, *in situ* deployment of traditional wired geophones is labor intensive for a dense sensor array. Alternatively, stand-alone seismometers can be used, but they are bulky, heavy, and expensive because they are usually designed for long-term monitoring. To better facilitate field applications of the passive surface wave survey, a low-cost energy-efficient geophone system was developed in this study. The hardware design is presented in this paper. To validate the system’s functionality, both laboratory and field experiments were conducted. The unique feature of this newly-developed cableless geophone system allows for rapid field applications of the passive surface wave survey with dense array measurements.

## 1. Introduction

Geotechnical site investigations often involve surface and subsurface explorations. For subsurface exploration, geotechnical engineers or engineering geologists usually conduct *insitu* tests, such as standard penetration tests (SPT) and cone penetration tests (CPT) to determine soil conditions. Soil samples collected through these tests are useful for visual observations and laboratory tests, which are essential tasks for detailed site investigations [1]. Geophysical techniques have been increasingly used as alternatives to such invasive tests in applications for solving geotechnical engineering problems during the past few decades. Although these techniques cannot completely replace direct physical tests, they can quickly and cost-effectively obtain preliminary subsurface information over large areas.

A variety of geophysical techniques can be used for geotechnical site investigations [2], including surface wave methods (SWMs), which use either artificially generated surface ground motions or ambient ground vibrations (microtremors) for subsurface geotechnical explorations. The development and technical basics of SWMs were summarized in Pelekis and Athanasopoulos [3] and Foti *et al*. [4]. Typically, the SWMs requiring artificially-generated surface waves are called active surface wave methods while those measuring microtremors are called passive surface wave methods. Typical active surface wave methods include the Steady State Rayleigh Waves [5], the Spectral Analysis of Surface Waves (SASW) [6], and the Multichannel Analysis of Surface Waves (MASW) [7]. The passive surface wave methods were initially developed in the 1950s as microtremor survey methods (MSM). Due to their nonintrusive nature, these methods have been incorporated into geotechnical engineering applications since the passive MASW [8] and the Refraction Microtremor (ReMi) [9] were developed. These applications are in the fields of seismic microzonation [10], liquefaction risk assessment [11], underground anomaly detection [12], site soil characterization [13], and underground structure surveys [14]. Commonly used passive surface wave methods for field data processing include the spatial autocorrelation (SPAC) method [15], the extended spatial autocorrelation (ESPAC) method [16], the frequency-wave number (FK) approaches [17], and the Horizontal-to-Vertical Spectral Ratio (HVSR) method [18].

Geophones are the most widely used signal receivers for passive surface wave surveys, although sometimes accelerometers are also used [19,20,21]. The core component of a modern motion sensing geophone is an inertial mass suspended from a spring with a magnet attached to the geophone case and a coil used as a proof mass. Geophones do not need any external electrical power supply, which is beneficial for field applications. A passive surface wave survey typically involves a number of commercial geophones (e.g., 12 or 24) that are wired together and connected to a seismograph through cables for data collection. If a dense receiver network is needed, these cables would introduce substantial complexity to the field instrumentation. Sometimes, vehicles may be required for high-density cable-based geophone deployment in oil and gas exploration. Stand-alone seismometers, which belong to broadband sensors, e.g., 0.03–50 Hz for the CMG-40 [22], are sometimes used as receivers for passive surface wave surveys. However, conventional broadband seismometers are expensive, bulky, and heavy, which limits the number of stations that can be deployed simultaneously and affects measurement efficiency.

Although efforts have been devoted to the development of unattended ground sensor systems [23,24], few studies have been reported on the advancement of passive surface wave survey instrumentation. A scheme using wireless technology has been proposed by Picozzi *et al*. [25]. However, this scheme, originally being developed for earthquake early warning systems for mega-cities, is more suitable for large-area deployment and long-term monitoring purposes. Picozzi *et al.*’s wireless system consumes a high amount of energy to operate, thus the system requires either a main power supply or solar panels for field applications [25]. Another challenge in using these systems is meeting the strict simultaneous measurement requirement between different receivers during a MSM field testing, e.g., time difference less than 2 ms and phase difference smaller than ±3°, if wireless communication and real-time analysis are implemented as suggested by Picozzi *et al.* [25].

The current study resulted in the development of a low-cost, energy-efficient geophone system for ambient-noise measurements, which allows a dense array receiver deployment for passive surface wave surveys. The general design philosophy and the laboratory validation tests of the newly-developed cableless geophone system are reported in the paper to demonstrate its functionality. In addition, field experiments have been conducted at a wind farm and an abandoned mine. The outcome of these experiments indicated that the passive surface wave method using cableless geophones is an effective and economic method for preliminary geotechnical engineering investigations.

## 2. Development of the Low-Cost Energy-Efficient Geophone Unit

### 2.1. Sensor Development

Motion-sensing geophones, based on an inertial mass suspended by a spring, are a commonly-used surface wave signal receivers for high-frequency range applications. Geophones have priority over other passive sensors because they are inexpensive. The novel cableless geophone system in this study was designed with attention to cost effectiveness, portability, energy consumption, and functionality. Several commercially-available geophones with different frequency bands (1 Hz, 2 Hz, 4.5 Hz, 10 Hz) [26] were considered. For comparison purposes in this study, a commercially-available stand-alone seismometer, CMG-40 [22], was used to evaluate the geophone performance. A critical parameter for selecting geophones is hardware noise, which is defined as the output signal with a zero ground motion input. If a geophone has relatively high hardware noise, it is impractical for use in measuring microtremor signals, which typically have a very small nominal peak amplitude (10^−10^–10^−2^ m/s) over the frequency range of 0.001–50 Hz [27]. In theory, hardware noise mainly comes from a vector combination of the Brownian circuit noise (Equation (1)) and the electronic thermal noise (Equation (2)):

(1) $${\bar{\text{F}}}^{2}/{\text{m}}_{\text{s}}^{2}/\text{Hz}={\text{a}}^{2}/\text{Hz}=\frac{8{\text{πK}}_{\text{b}}{\text{T}}_{\text{e}}}{{\text{QT}}_{0}{\text{m}}_{\text{s}}}$$

where ${\text{ K}}_{\text{b}}$ is the Boltzmann constant, ${\text{T}}_{\text{e}}$ is the temperature in Kelvin, Q is the inertial mass quality index, ${\text{T}}_{0}$ is the natural period of the inertial mass, and ${\text{m}}_{\text{s}}$ is the weight of the inertial mass.

(2) $${\text{V}}_{\text{n}}^{2}=4{\text{K}}_{\text{b}}{\text{T}}_{\text{e}}\text{RB}$$

where  R is resistance value, and B is the frequency band.

**Figure 1:**
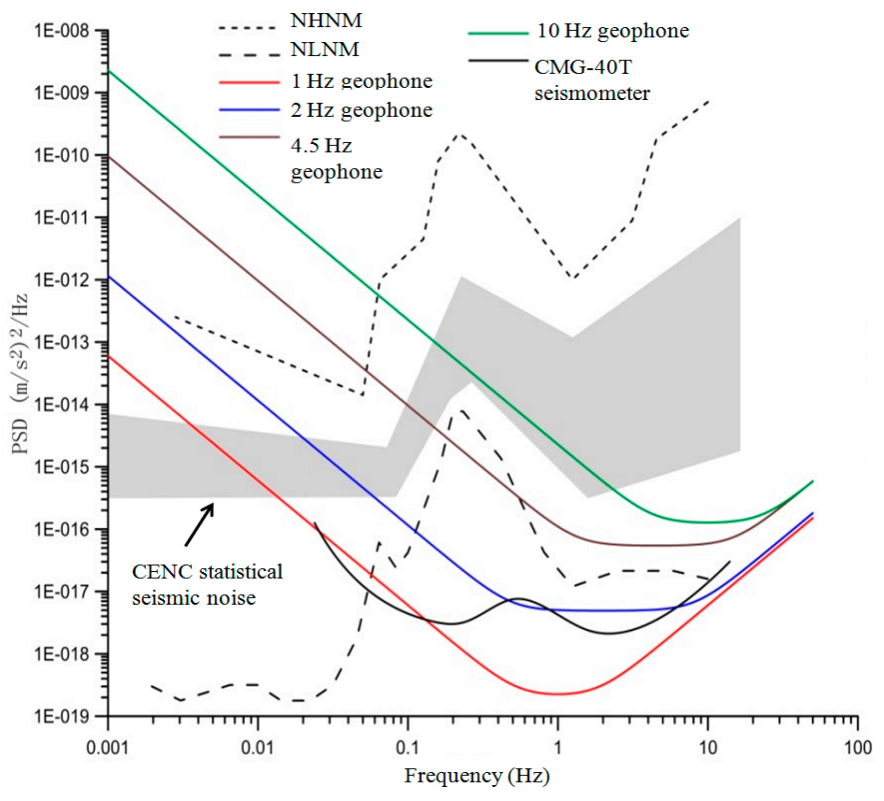
Power spectrum density comparison of four geophones (1, 2, 4.5, and 10 Hz) and the CMG-40T seismometer.

**Figure 2:**
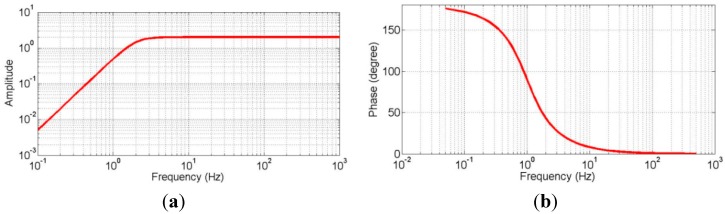
Amplitude and phase responses of the 2-Hz geophone. (**a**) amplitude response; (**b**) phase response.

Power spectrum densities (PSD) of hardware noise for the four candidate geophones and the CMG-40T seismometer were calculated using Equations (1) and (2). Figure 1 compares the calculated hardware noise PSDs with the Perterson curves [28], including the new high-noise model (NHNM) and the new low-noise model (NLNM) [28]. In addition, the seismic noise statistical records (shaded area) from the China Earthquake Networks Center (CENC) were also digitized and marked in the figure. Based on Figure 1, this study made the following observations: (1) the geophone hardware noise increases as the frequency of the tested vibration decreases; (2) the statistical seismic noises of China have higher PSDs than that of the NLNM curve overall frequencies, which indicates that a candidate geophone with hardware noise below the NLNM curve should work well in collecting microtremor signals in the Chinese geological environment; and (3) the hardware noise PSDs of the 1-Hz and 2 Hz geophones as well as the CMG-40T are below the NLNM curve at the frequency range of 0.2–10 Hz. Therefore, the 1 Hz and 2 Hz geophones are suitable for geotechnical engineering applications of the passive surface wave methods. Although it is superior to the 2 Hz geophone in terms of hardware noise at lower frequencies, the 1 Hz geophone comes with a huge increase in hardware cost and size. Therefore, the 2 Hz geophone was finally selected as the sensing unit of the cableless geophone system design in this study. Table 1 lists the technical specifications of the selected 2 Hz geophone, and its amplitude and phase responses are shown in Figure 2.

Typical microtremor signals have low vibration amplitudes, thus a compatible operational amplifier (OA) is required to work with the 2 Hz geophone to provide manageable outputs. However, the OA will add extra hardware noise to the cableless geophone system. The hardware noise of the geophone comes mainly from the voltage noise (Equation (1)) while that of the OA comes mainly from the electronic thermal noise (Equation (2)). An OA is acceptable if its hardware noise is smaller than that of the geophone, hence the total combined hardware noise of the cableless geophone system will not increase dramatically. The hardware noise levels of three commercially-available OAs were compared with that of the 2 Hz geophone in this study. A widely used general purpose amplifier, Texas Instruments UA741 [29], was found unacceptable for this study’s cableless geophone system because the amplifier’s noise is three times higher than that of the 2 Hz geophone. Linear Technology LT1028 [30] and Analog Devices OP27 [31] are good candidates because their hardware noise levels are approximately 10% and 33% of the 2 Hz geophone hardware noise, respectively. Compared with LT1028, OP27 uses much less energy (50% less), which can be beneficial for field applications. Therefore, the OP27 was finally selected to work with the 2 Hz geophone for the cableless geophone system design.

**Table 1:** Technical specifications of the selected 2-Hz geophone [26].

Description		Value/Range
Natural frequency (Hz)		2% ± 10%
Sensitivity (V/cm/s)		2% ± 10%
Coil resistance (Ω)		6040% ± 5%
Open circuit damping		0.7% ± 10%
Harmonic distortion		≤0.2
Insulation resistance (MΩ)		≥20
Maximum coil travel (mm)		3
Inertial mass weight (g)		60
Operation temperature (°C)		−25~+55
Weight (g)		250
Size (mm)	Diameter	38.5
	Height	47

Analog-to-digital (A/D) convertors are commonly used to support multichannel and high-frequency rate sampling in seismometers. An A/D converter has been adopted in the study’s cableless geophone unit design to digitize the microtremor signals collected by the 2 Hz geophone. Popularly used commercial A/D converters include Analog DevicesAD7766-2 [32], Texas Instruments ADS1281/2 [33], and Cirrus Logic CS5371A [34]. These A/D converters are expensive and use a large amount of energy because they are primarily designed for multichannel data acquisitions. Since the 2 Hz geophone in this study is a uniaxial receiver, Texas Instruments ADS1251 [35] was selected to reduce the total cost. ADS1251 has a sufficient sampling rate and is relatively energy efficient, which satisfied one of the design goals of this work.

**Figure 3:**
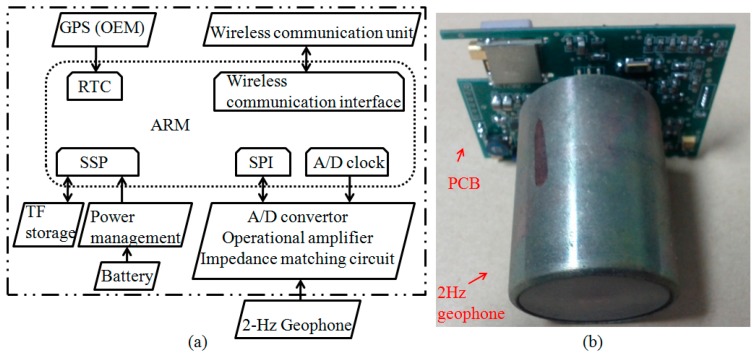
The cableless geophone system: (**a**) Design scheme, and (**b**) integrated unit with the printed circuit board (PCB) and the geophone (see the text for details about the various components).

The overall design scheme of the cableless geophone system is sketched in Figure 3. An advanced RISC Machine (ARM), LPC11U24 [36], is used to control all built-in hardware components, including a Real-Time Clock (RTC), the Synchronous Serial Port (SSP), the Serial Peripheral Interface (SPI), and an A/D clock. As shown in Figure 3, the 2 Hz geophone, as the sensing unit, collects microtremor data, which are then converted into digital signals through the A/D convertor. These signals are then timed by the A/D clock and stored in a TransFlash card (TP) (SanDisk 16G). A GPS unit, SR100 [37], is adopted for time and location management of the cableless geophone system. A wireless communication unit can also be added by an integrated design of the ARM. With the added wireless communication unit, triggering the sensor and retrieving signal records wirelessly can be easily implemented. The entire system is powered with a 3.7 V and 950 mAh Lithium-ion battery (PisenTS-MT-G808) since it is not designed for long-term monitoring.

With the current design version, the total weight of the prototype system is only 600 g, which is much lighter than that of most stand-alone seismometers (>10 kg). This unit also operates with superior energy efficiency; the maximum power consumption is only 100 mW when the system is fully operating and 1 mW when the system is in sleep mode. Another noteworthy advantage of this cableless geophone unit is its cost efficiency. A single unit, with mass production, costs less than $600 U.S. dollars, while the stand-alone seismometers in market typically cost more than $13,000 U.S. dollars per unit).

### 2.2. Laboratory Calibration

**Figure 4:**
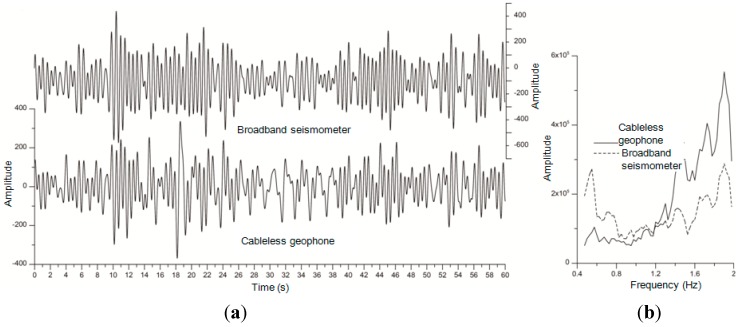
Comparison between the seismometer measurements and the cableless geophone records: (**a**) time histories; and (**b**) frequency spectra.

Laboratory experiments were conducted to validate the functionality of the newly-developed geophone unit. In the first lab experiment, one cableless geophone system was tested and compared with a seismometer at a seismograph station of the China Earthquake Network. A typical test record comparison presented in Figure 4 for a 60 s window. The frequency spectra of the two records were obtained by the Fast Fourier Transform (FFT) processing. Due to the hardware difference and the impossibility of placing both sensors at the same spot, these two records are not exactly the same. However, they show a relatively good correlation for frequencies between 0.8 and 1.4 Hz. Figure 4 also indicates that the cableless geophone system performs better than the seismometer for frequencies greater than 1.4 Hz. However, the cableless geophone unit’s performance is not as good as the seismometer in the low frequency range (0.4–0.8 Hz), which represents a deep underground exploration case. In conclusion, the cableless geophone system, with a workable frequency band containing frequencies even lower than 1 Hz, should be sufficient for measuring depths within a couple of hundred meters below the ground surface, the depth that most civil engineers are interested.

**Figure 5:**
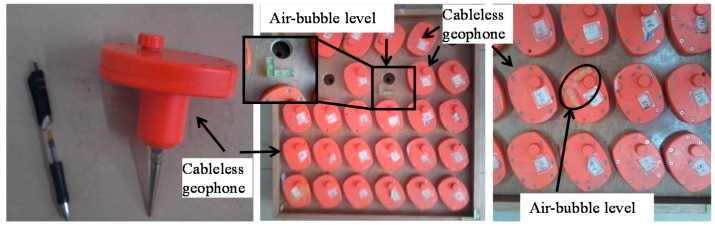
Cableless geophone correlation test setup.

**Figure 6:**
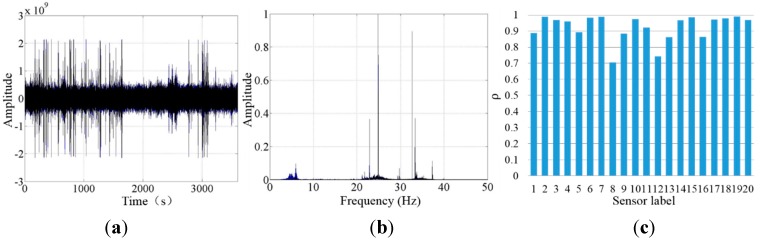
Cableless geophone correlation testing results: (**a**) amplitude time series collected by 21 sensors; (**b**) power spectral densities of the signals; and (**c**) correlation coefficients between different sensors.

The second laboratory experiment was designed with a total of 21 units placed on a specially-developed platform (Figure 5) to validate the synchronization. The levels of these geophone units were calibrated by two air bubble levels. With a sampling rate of 100 Hz, these 21 units were simultaneously collecting ambient noise in a controlled environment. The test was performed from 24:00 midnight to 1:00 a.m. continuously. Time histories collected by 21 sensors and their power spectral densities are shown in Figure 6. As indicated in the figure, these 21 sensors work properly and they provide close measurements in both time and frequency domains. A correlation coefficient (ρ) (Equation (3)) was used to compare the amplitudes of time history signals collected by these 21 geophone systems. It was found that most cableless geophone systems showed relatively good correlation performances (Figure 6) with correlation coefficients larger than 0.8.

(3) $$\text{ρ}=\frac{\text{E}\left[\left(\text{x}-{\text{μ}}_{\text{x}}\right)\left(\text{y}-{\text{μ}}_{\text{y}}\right)\right]}{{\text{σ}}_{\text{x}}{\text{σ}}_{\text{y}}}$$

where E is the expectation value; ${\text{μ}}_{\text{x}}$ and ${\text{μ}}_{\text{y}}$ are the mean of two signals, x and y, respectively; ${\text{σ}}_{\text{x}}$ and ${\text{σ}}_{\text{y}}$ are the standard deviation of these two measurements.

## 3. Field Tests for Validation and Demonstration

### 3.1. Array-Based Passive Surface Wave Survey Method

A trait of surface waves is that their vibration displacement decays exponentially with depth. Also the penetration depth of a surface wave depends on its wavelength. This trait has been used to characterize site soil properties. In practice, most surface wave applications generate a shear-wave velocity profile until a certain depth by using a three-step strategy: (1) acquiring experimental data; (2) obtaining the experimental dispersion curve through signal processing; and (3) solving an inverse problem with a reference model.

Array-based passive surface wave surveys are typically conducted by deploying a group of receivers on the surface to collect ambient vibrations created by either natural events or human activities close to the site of interest. Receivers can be located as a linear or array layout. The maximum dimension of the array is usually related to the measurement depth. With the cableless geophone system developed in this study, surface wave surveys can be performed over a denser spatial array, which not only allows a much greater range of spatial lags to be sampled in less time, but also enhances the accuracy of the shear wave velocity profile estimation.

### 3.2. Field Testing at a Wind Farm

**Figure 7:**
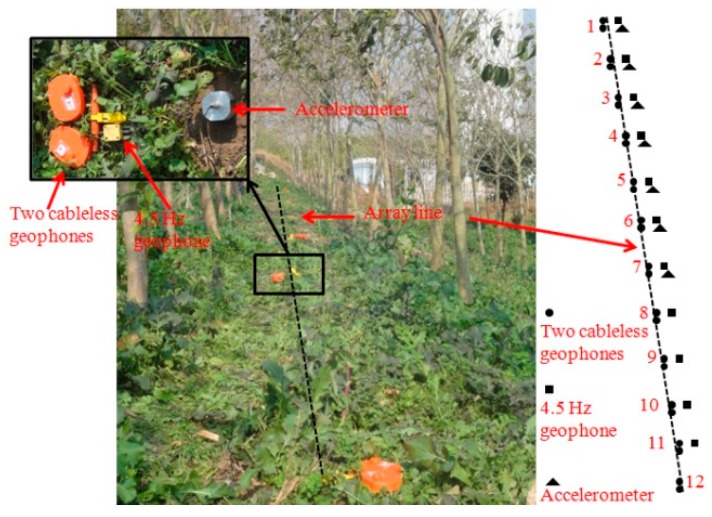
Receiver deployment for the wind farm field testing.

The first field validation test was conducted at a wind farm. Because of geographical space limitations, a linear layout (1-D) of the receiver network was adopted, with the direction pointing to the excitation source, a wind turbine tower. Three different types of receivers were employed on the site: (1) wired integrated circuit piezoelectric accelerometers; (2) the newly developed cableless geophone systems; and (3) conventional 4.5 Hz wired geophones (Figure 7). Technical specifications of the wired accelerometer and the 4.5 Hz geophone are listed in Table 2 and Table 3, respectively.

**Figure 8:**
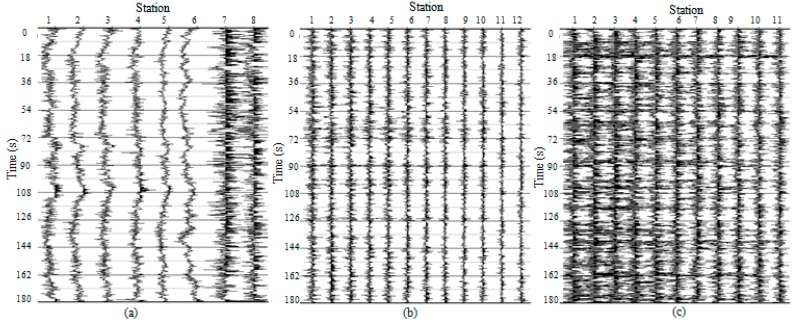
Typical time history series from: (**a**) accelerometer; (**b**) cableless geophone; and (**c**) 4.5 Hz geophone.

A total of 12 stations were deployed at an interval of 5 m. Two cableless geophone systems were placed side by side at each station. One wired accelerometer and one wired 4.5 Hz geophone were placed close to the two cableless geophone systems. Four of the 12 wired accelerometers and one of the 12 wired 4.5 Hz geophones did not function properly during the field test. Therefore, a total of 11 wired geophones and eight wired accelerometers collected data, as shown in Figure 8. The wired 4.5 Hz geophones acquired data by a seismograph. Also a data acquisition system and a laptop were used to collect accelerations from the wired accelerometers. The vibration data collected by the cableless geophone units were directly stored in the TransFlash card inside each unit. The data were retrieved after the completion of the field experiments. Three 20 min microtremor measurements were conducted at the site with the sampling rate set to 200 Hz.

**Table 2:** Technical specifications of the wired accelerometer.

Technical Specifics		Accelerometer
Sensitivity		40,000 mV/g
Measurement range		0.12 g
Frequency band		0.5–1000 (±10%) Hz
Resonant frequency		3 kHz
Resolution		0.0000005 g
Operation temperature		−40~+120
Weight		310 gm
Size	Diameter	45 mm
	Height	30.5 mm

**Table 3:** Technical specifics of the wired 4.5 Hz geophone.

Description		Value/Range
Natural frequency (Hz)		4.5% ± 10%
Sensitivity (V/cm/s)		0.28% ± 5%
Coil resistance (Ω)		365% ± 5%
Open circuit damping		0.7% ± 10%
Harmonic distortion		≤0.2
Insulation resistance (MΩ)		≥20
Maximum coil travel (mm)		2
Inertial mass weight (g)		8.5
Operation temperature (^o^C)		−40~+75
Weight (g)		85
Dimension (mm)	Diameter	27.2
	Height	34

**Figure 9:**
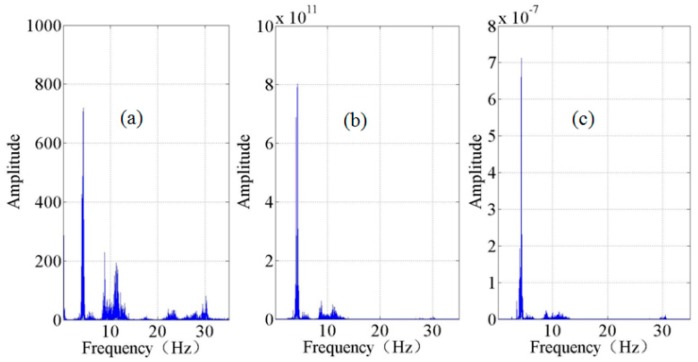
Typical recordings in the frequency domain: (**a**) accelerometer; (**b**) cableless geophone; and (**c**) 4.5 Hz geophone.

Typical 180-s time series and their corresponding spectra are shown in Figure 8 and Figure 9, respectively. These time series/spectra are acceleration from the accelerometers and velocity from both the wired geophones and cableless geophone systems. The waveforms in Figure 8 and the power spectral densities in Figure 9 indicate that the signals collected by the accelerometers contain more low frequency (<1 Hz) and high frequency (>20 Hz) components than those collected by the geophones and cableless geophone systems because of the higher sensitivity and broadband feature of accelerometers.

**Figure 10:**
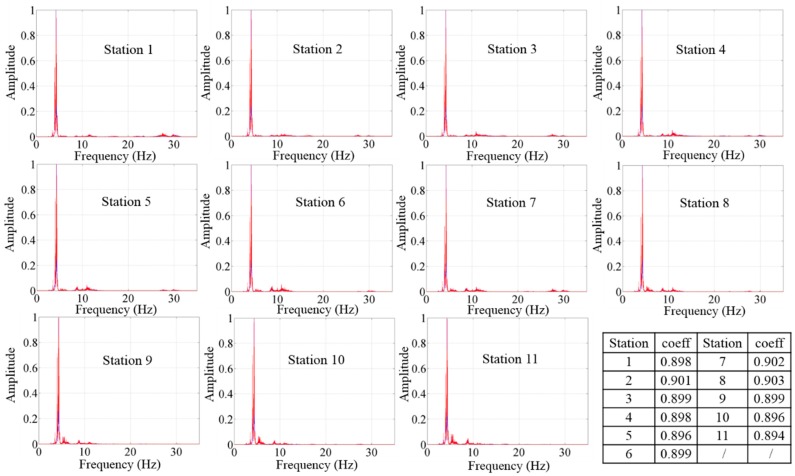
Power spectral densities of signals collected from the cableless geophone (red) and the 4.5 Hz geophone (blue) and the correlation coefficients for each station.

The data collected from the new cableless geophone system was compared with that obtained by the 4.5 Hz geophone for validation purposes. It should be pointed out that the 4.5 Hz wired geophone is considered a widely used “standard” receiver in surface wave survey practice. Since the cableless geophone system and the 4.5 Hz geophone were not synchronized during the field testing, the data were compared in the frequency domain. A total of three datasets were considered for the comparison. The comparison of the power spectral densities of the data through the two sensors, *i.e.*, the new cableless geophone and the 4.5 Hz wired geophone, are presented in Figure 10 for a total of 11 stations, where both the developed system and the 4.5 Hz geophone worked properly. The correlation coefficients in Figure 10 were calculated using Equation (3) for each station. The average of these correlation coefficients was around 0.9, which indicates that the data obtained from the new cableless system have a comparable quality with that collected by the conventional wired geophone.

**Figure 11:**
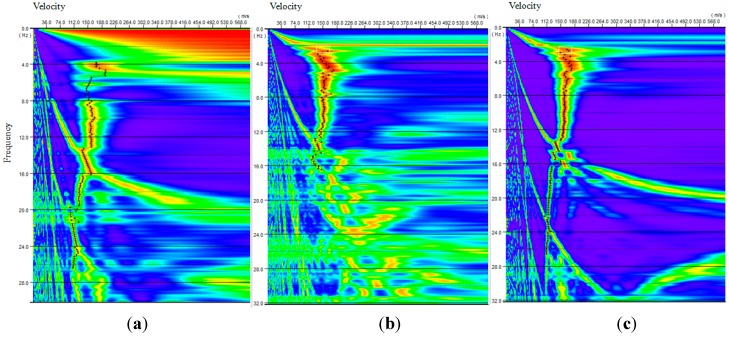
Dispersion images for recordings from: (**a**) accelerometer; (**b**) cableless geophone; and (**c**) 4.5 Hz geophone.

**Figure 12:**
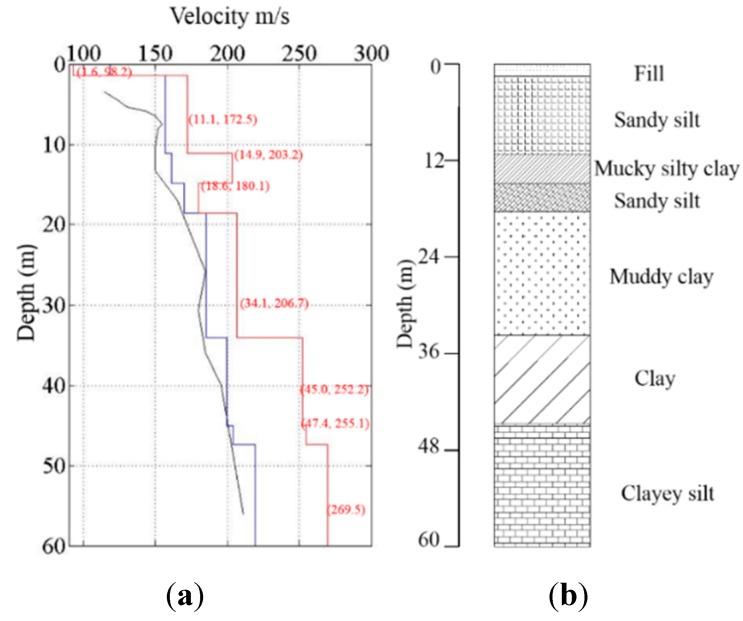
Shear wave profile of the test site: (**a**) shear wave velocity profiles; and (**b**) borehole soil profile. (The blue line is the initial shear wave velocity and the red line represents the final velocity profile.).

Time series recordings from three different types of receivers were then processed by using the FK method [17] for dispersion images (Figure 11). In the result analyses, three 20-minute signals for each type of sensor were stacked to improve the data quality. Dispersion curves were further extracted from the dispersion images. Finally, shear wave velocity profiles of the tested site were obtained by the inversion analyses of these dispersion curves. Figure 12 shows the shear wave velocity profile of the wind farm site, compared with a previous borehole test result. In the figure, the blue line represents the initial shear wave velocity in the genetic analysis (GA) inversion processing and the red line represents the final shear wave velocity profile. It should be noted that the testing region has a thick layer of alluvial soil as reported in other studies [38]. Due to the limitations on the length of the receiver layout line and the sensitivity of the hardware (2 Hz or 4.5 Hz geophone), the shear wave velocity profile is reported only down to 60 m, which is still within the alluvial soil layer. Therefore, the shear wave velocity in the profile does not show dramatic changes. However, the resulting velocity values match the actual soil properties at this site, which yields an average velocity of 209.8 m/s. This field validation test indicates that the new system functions well for passive surface wave survey applications.

### 3.3. Field Testing at an Abandoned Coal Mine

The second field validation experiment was conducted at an abandoned coal mine area in East China. This underground coal mine, which was fully operational in the 1950s, has been abandoned for decades. The local government has been considering this area for new building construction. Therefore, geotechnical information has to be obtained for site assessment and foundation design.

**Figure 13:**
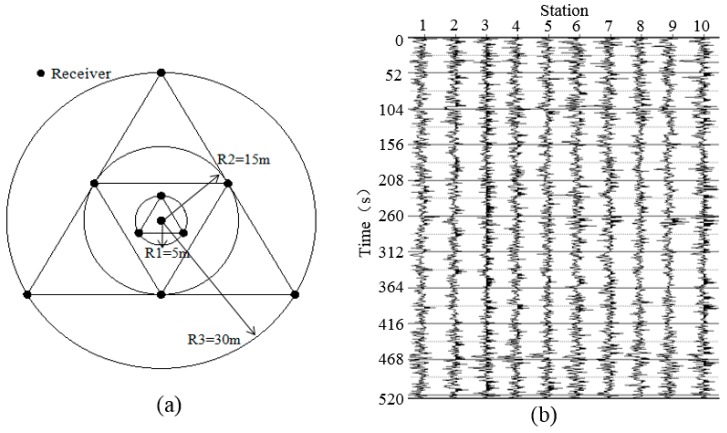
Test at an abandoned mine: (**a**) array design; and (**b**) typical vibration recordings.

The passive surface wave survey was adopted as a preliminary investigation strategy before borehole drillings are conducted. To facilitate this *in situ* surface wave survey, the new cableless geophone systems were used as receivers, which were deployed on three circles with the same center point as shown in Figure 13. The diameters of the three circles are 10 m, 30 m, and 60 m. On each circle, three cableless geophone systems were deployed forming an equilateral triangle shape. An additional cableless geophone system was placed at the center point of these three circles. Therefore, 10 cableless geophone systems were used. Through this receiver layout, geotechnical information can be estimated for the area covered by the 60-m diameter circle.

**Figure 14:**
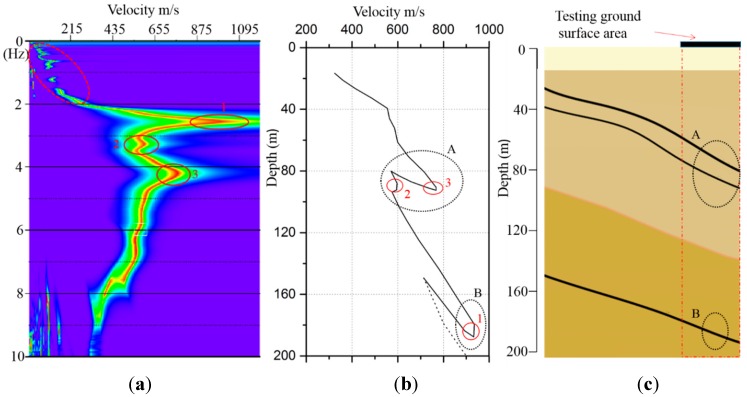
Coal layer estimation: (**a**) dispersion image; (**b**) velocity profile; and (**c**) geological profile data.

During the tests, the 10 cableless geophone units collected seismic noise for 30 min simultaneously, with the sampling rate set to 100 Hz. Typical ambient noise time series records of 520-s from the 10 units are shown in Figure 13. These records were stacked and processed using the SPAC method [15], which resulted in a dispersion image as shown in Figure 14a. Before being processed, obvious abnormal signals were filtered out. The abrupt velocity change points (solid line circles labeled as 1, 2 and 3) were associated with the dramatic changes of soil properties as reported in other studies (e.g., [39]). It should be noted that the sensing unit of the new cableless geophone system is a 2-Hz geophone, whose transferring function has a plateau for frequencies beyond 2 Hz (Figure 2). Theoretically, the recording bandwidth should be above 2-Hz for a commonly damped system [40]. By using the compatible OA and the A/D convertor in the current design, the new cableless geophone system can still work reasonably well for frequencies at 1-2 Hz (Figure 4). However, caution is required in field practices in order to obtain meaningful results when using data over this low frequency band. In this case, dispersion results in the low frequency range in Figure 14a (the dashed line circle) are misleading because of the limitations of hardware and the receiver layout diameter. Therefore, the tested data below 2 Hz cannot be acceptable for the shear wave velocity profile analysis. The depth at the abrupt velocity change locations was estimated by using the dispersion curve directly as shown in Figure 14b. Since the dispersion results below 2 Hz were not considered when processing the tested data, the dash line of the velocity profile in Figure 14b was simply estimated based on engineering judgment that the phase velocity usually increases with the increasing of depth underground for a common geological setting. As shown in Figure 14b, two obvious velocity change regions (circles “A” and “B”) are located at around 80 m and 180 m below the surface, respectively. This estimation agrees well with the geological information obtained from the borehole testing (Figure 14c), which shows three underground coal layers. The first layer is located at about 70 m below the surface, while the second and third layers are located approximately at depths of 80 m and 180 m, respectively. Since the first and second layers are too close to each other, the field surface wave survey was unable to distinguish them. In general, the *in situ* surface wave surveys can provide a good estimation of the depths of underground mined coal seams based on wave velocity changes. Of course, the depth resolution can be improved by using a denser receiver deployment; deeper coal layers can also be detected by using a larger sensor deployment circle. Please note that the correlation between the abnormal velocity change or the so-called “zigzag” phenomenon in the dispersion curve and the low-frequency zone in a layered media is a complex problem in theory. Please refer to Tokimatsu *et al.* [41] and Lu and Zhang [42] for details. However, previous studies have proved (e.g., [39]) that the empirical method using the inflection points in the dispersion curve to estimate the low-velocity layer position is acceptable in engineering practices.

## 4. Conclusions

A low-cost energy-efficient cableless geophone system was designed and developed for ambient-noise measurements, which allows a dense array to be deployed for field surface wave surveys. Hardware components of the unit were selected from commercially available products based on a comprehensive consideration of cost effectiveness, portability, energy consumption, and functionality. A 2-Hz geophone was finally adopted as the sensing unit in the cableless geophone system design because of its reasonable price and suitable sensitivity within the frequency range of interest. Since the seismic noise signals typically have a low vibration amplitude, a compatible operational amplifier, OP27, and an analog-to-digital convertor, ADS1251, were attached to the 2-Hz geophone in order to provide readable outputs. An ARM, LPC11U24, with a built-in RTC, a SSP, a SPI, and an A/D clockworks as the controller of the cableless geophone system. A local storage strategy was implemented in the design by using a 16G TransFlash card. A SR100GPS unit was adopted for time and location management of the cableless geophone system. With the current design version, the maximum power consumption for the entire unit is only 100 mW for full operation and 1 mW in sleeping mode. A prototype cableless geophone system weighs only 600 g and costs less than 600 U.S. dollars.

Two laboratory experiments were conducted to validate the functionality of the new geophone units. The waveform comparison between an in-service broadband seismometer and the unit proved that the cableless geophone system can effectively collect ambient ground vibration signals over a frequency bandwidth suitable for geotechnical engineering survey purposes. Additionally, a correlation test for a group of 21 units was conducted in a laboratory. These cableless geophone systems showed reasonable correlation performances for array-based surface wave survey applications. 

Two *in situ* tests were then conducted for field validation of the new geophone units. For the first field test, three different types of receivers were employed for a wind farm geotechnical survey. Ambient ground vibration signals were collected and processed to obtain shear wave velocity profiles for the site down to 60 m below the surface. The cableless geophone systems showed superior performance over the conventional 4.5 Hz wired geophones. For the second field test, an array-based surface wave survey with the cableless geophone units serving as receivers was implemented at an abandoned coal mine for preliminary geotechnical investigation. Microtremor measurements from the units were successfully used to estimate the underground mined coal seam depths based on shear wave velocity changes. These two practical cases demonstrated the effectiveness and functionality of the newly developed cableless geophone systems for geotechnical surveys.

It is expected to promote the application of this cableless geophone system through more field testing demonstrations in the near future. Efforts are continuously devoted to upgrade the system for various application purposes, such as a large scale dense deployment for monitoring and deep underground exploration.
